# Electrophysiological Regulation of Nutrient Transport in Mangrove Species Under Salinity Stress: A Comparative Physiological Analysis of *Aegiceras corniculatum* (L.) Blanco and *Kandelia obovata* Sheue, H.Y. Liu & J.W.H. Yong

**DOI:** 10.3390/plants14203228

**Published:** 2025-10-20

**Authors:** Kashif Ali Solangi, Yun Wang, Yanyou Wu, Mazhar Hussain Tunio, Farheen Solangi, Irfan Abbas, Jinling Zhang, Xiqiang Song

**Affiliations:** 1 Hainan Institute of National Park/Key Laboratory of Conservation and Development of Hainan National Park, Haikou 570203, China; kashifsolangi@hainanu.edu.cn; 2 Key Laboratory of Genetics and Germplasm Innovation of Tropical Special Forest Trees and Ornamental Plants (Ministry of Education), School of Tropical Agriculture and Forestry, Hainan University, Danzhou 571700, China; 18889519790@163.com; 3 Key Laboratory of Modern Agricultural Equipment and Technology, Ministry of Education, Institute of Agricultural Engineering, Jiangsu University, Zhenjiang 212013, China; mazharhussaintunio@sau.edu.pk (M.H.T.); dr.iabbas@yahoo.com (I.A.); 4 State Key Laboratory of Environmental Geochemistry, Institute of Geochemistry, Chinese Academy of Sciences, Guiyang 550081, China; yanyouwu@mail.gyig.ac.cn; 5 Research Centre of Fluid Machinery Engineering and Technology, Jiangsu University, Zhenjiang 212013, China; dr.farheensolangi@gmail.com

**Keywords:** mangrove mechanisms, salt resistance capacities, ion homeostasis, salt stress, growth rate model

## Abstract

Salinity is a major environmental constraint that influences nutrient acquisition and internal transport in coastal plant species. However, the electrophysiological mechanisms underlying nutrient flow regulation in mangroves remain poorly understood. This study investigates the active transport flow of nutrients (NAF) and nutrient plunder capacity (NPC) in two ecologically significant mangrove species, *Aegiceras corniculatum* (L.) Blanco (*A. corniculatum*) and *Kandelia obovata* Sheue, H.Y. Liu & J.W.H. Yong (*K. obovata*), using intrinsic electrophysiological leaf traits including inherent impedance (IZ), inherent capacitive reactance (IXC), inherent inductive reactance (IXL), and inherent capacitance (IC). A randomized block design was employed using three different saline treatments with control, such as control (0 mM), low (T1,100 mM), medium (T2, 250 mM), and high (T3, 450 mM). The results of the fitting equations show a positive correlation between resistance (Re), capacitive reactance (XC), and inductive reactance (XL) with clamping force (CF); all values of R^2^ are ≥0.98, and *p*-values are <0.0001. Nutrient transport capacity (NTC) was non-significant in control and low-salt treatment for both mangrove species, indicating resistance to low levels of salt stress. NAF results of *A. corniculatum* showed a slight reduction of 7.9% under low salinity, while *K. obovata* displayed strong positive responses NAF increasing by 63.7% compared to the control. Additionally, the NPC of *A. corniculatum* species was not significantly affected at low and medium salt levels but declined at high salt levels. In contrast, *K. obovata* exhibited a higher growth rate and better photosynthetic performance than *A. corniculatum*. Our findings provide novel mechanistic insights into how electrophysiological regulation governs nutrient transport under salinity stress and highlight interspecies differences in adaptive strategies, with implications for understanding mangrove resilience in saline environments.

## 1. Introduction

Mangroves are the most productive ecosystems on earth, found in the intertidal zone of tropical and sub-tropical coastal regions [1,2,3]. The coastal ecosystem is beneficial for humans and provides basic needs of life such as food, raw materials, timber, tourism, and herbal medicines [4]. Due to rapid urbanization and industrialization, these are at high risk and are increasing day by day [5]. More than one-third of the world’s population, directly or indirectly, depends on wetlands [3,6]. In earlier years, a lot of industrial, agricultural, and sanitary sewage had been concentrated in estuaries and gulf regions [7]. Because of this large amount of nitrogen (N) and phosphorus (P) entering the coastal ecosystem and increasing the eutrophication, this is also a main reason for the decline in water quality in coastal areas [8,9]. The restoration of disturbed wetlands is an urgent issue concerning the sustainable development of coastal ecosystems and expands the attention of previous research studies [5,10,11]. Therefore, a dynamic plan is needed for the management of mangrove species according to local conditions and increased mangrove population based on their mechanisms, nutrient plunder capacity, and salt resistance capacity, which helps the restoration of mangrove forests as well as the coastal ecosystem.

Mangroves play a very important role in the coastal areas and help to develop these areas with their unique features [12]. Mangroves also provide a vast range of economic and ecological benefits for agro-ecosystems [2]. Mangroves could minimize the salinity level and significantly reduce the effect of seawater intrusion on coastal areas [13]. Recent research evaluated the response of two different mangrove species based on electrophysiological parameters at different salt levels. In this study, it was observed that during the stress condition, cell volume plays an important role in resisting the salt stress [4]. Further, it is also notified that *Kandelia obovata* Sheue, H.Y. Liu & J.W.H. Yong (*K. obovata*) can store more salt in high-stress conditions [14,15]. *K. obovata* species do not secrete their salt because all salt ions are present in the vacuole, but the accumulation of plastoglobuli is an important strategy employed by *K. obovata* against salt stress [16,17]. In contrast, *Aegiceras corniculatum* (L.) Blanco (*A. corniculatum*) relies more on salt secretion through specialized salt glands present on its leaf surface, thereby preventing excessive ionic buildup within tissues. This mechanism highlights its adaptive strategy to regulate ion homeostasis under saline environments. These mechanisms help mangroves to resist salt stress but do not state their nutrient ion capacity, which plays an important role in sustaining growth and resilience across coastal regions [18]. The membrane lipid is an important element of the cell membrane, and it could be acting as an insulating layer along with electrical resistance, which allows the plant cell to store the electric charge [19]. Additionally, about 95% of the water in plant leaves evaporates through transpiration, leaving the remaining 5% of water in the leaf cell available to support the plant’s growth [20]. This 5% of valuable intracellular water is used for metabolism, which performs an important physiological function [21]. Plant cells at the time underwent biotic or abiotic stress, causing changes in the structure and makeup of ion permeability, which led to changes in their electrical characteristics.

A previous research study reported that different plants have different desalination effects under stressful environments [22]. To understand the desalination effects, we first need to understand the involvement of plant life activities. Photosynthesis parameters such as net photosynthesis (PN), leaf stomatal conductance (LSC), leaf intercellular CO_2_ concentration (LCi), and leaf transpiration rate (LTr) are highly sensitive to high salinity stress, showing marked reductions under such conditions. These physiological parameters directly influence essential plant life processes, particularly the regulation of leaf water content (WC) and the maintenance of cellular turgor and volume. In the current study, photosynthesis parameters are utilized as supportive parameters. Moreover, electrophysiological parameters play a vital role and deliver basic and important knowledge of plant life activities through the plant cell and water status. Electrophysiological parameters such as physiological capacitance (CP), resistance (Re), and impedance (Z) play an important role in the function of plant life, such as water metabolism, salt stress resistance, signal transduction, and energy formation. Because of the abiotic or biotic stress, plant cells are damaged, and observed changes in composition, structure, and ion permeability are evoked instantaneously, resulting in significant changes in their electrical parameters [23]. In our previous study, we successfully monitored the electrical parameters of two different mangrove species and checked the impact of re-watering and sodium nitroprusside (SNP) application. The outcome of the study provides significant information on water status and salt transport parameters in plant leaves, which could be determined rapidly, accurately, and in a simple way with the help of electrophysiological parameters [24].

Generally, the minimum concentration required for ion absorption, as determined by ion absorption kinetics, directly reflects a plant’s nutrient plunder capacity (NPC), that is, its ability to actively absorb and transport ions under low external concentrations. However, it cannot be used for in situ online detection, and the operation is complex, time-consuming, and labor-intensive [20]. The stronger a plant’s NPC, the stronger its affinity for ions, and the higher the concentration difference between the inside and outside of plant cells. The evaluation of the NPC of mangrove species has not been reported. The ratio of capacitive reactance (XC) and inductive reactance (XL) can represent the relative plunder information and active transport information. In this context, the objective of the present study was to establish a novel electrophysiological approach to monitor the active transport flow of nutrients and quantify the NPC of two mangrove species, *A. corniculatum* and *K. obovata*, under varying salt concentrations using mechanical equations.

## 2. Materials and Methods

### 2.1. Study Area and Experimental Design

A greenhouse experiment was performed at Jiangsu University, Zhenjiang, Jiangsu, China, with a latitude of 32.20 °N and a longitude of 119.45 °E, from March to May in 2023. The climate features of the district are humid and subtropical, with an average monsoon precipitation of 1058.8 mm per year and a mean annual temperature of 15.5 °C [25]. During the study period, the maximum, minimum, and mean temperature data, along with rainfall records, are provided in the Supplementary materials (Table S1 and Figure S2). One-year-old seedlings of two different mangrove species were collected from Quanzhou Tongqing Mangroves Technology Co. Ltd., Quanzhou City, Fujian Province, China. At first, both species were washed with tap water and stored in a 10 L, half-strength Hoagland solution for seven days [26]. A completely randomized design was chosen with five repeats in each treatment, and the experiment design is shown in Figure S1. Each pot contains 10 L of distilled water with a half-strength Hoagland solution. Three different sodium chloride (NaCl) treatments were used with a control treatment to examine the active transport flow of nutrients and the nutrient plunder capacity of two mangrove species, *A. corniculatum* and *K. obavata*. The treatments are as follows: control (0 mM), low (T1, 100 mM NaCl), medium (T2, 250 mM), and high (T3, 450 mM).

### 2.2. Data Collection

The data were recorded at every other day interval under the following parameters: leaf width and height were measured using a measuring scale for the estimation of growth rate (GR). Photosynthetic parameters such as net photosynthesis (PN), leaf stomatal conductance (LSC), leaf intercellular CO_2_ concentration (LCi), and leaf transpiration rate (LTr) were measured at three-day intervals at each sampling time, and every replicate was measured from the top of a fully planted young leaf. Further details of photosynthetic parameters are mentioned in Section 2.6. Although on a weekly basis, electrical parameters were also measured during the entire measurement, with the leaf attached to the plant.

### 2.3. Determination of Electrophysiological Parameters

**Figure 1:**
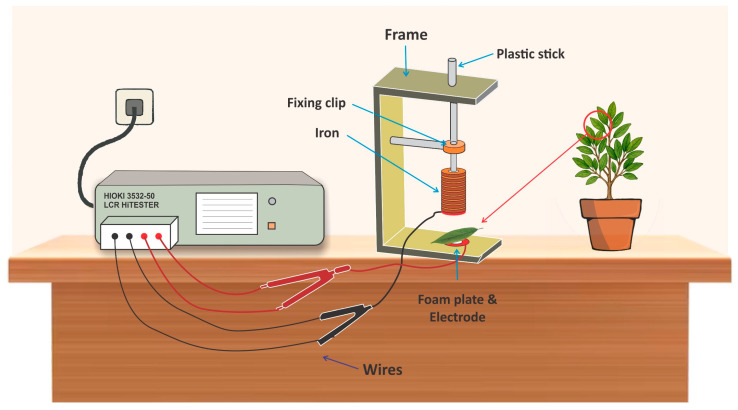
Parallel-plate capacitor setup connected to the LCR tester for real-time measurement of mangrove leaf electrophysiological parameters [24].

The variation in leaf capacitance (C), resistance (Re), and impedance (Z) at increasing clipping force (CF) was measured using an LCR tester (3532–50, HIOKI, Ueda, Nagano Prefecture, Japan). An LCR tester, also known as an LCR meter, is an electronic device used to measure the inductance (L), capacitance (C), and resistance (Re) of electronic components. The voltage (V) and frequency were 1 V and 3 kHz, respectively [24]. Generally, 1 V is used to prevent damage to plant tissue and to guarantee that measurements are made within the physiological tolerance of the plant cells. Higher voltages can lead to electroporation, which can harm the cell membrane and change its physiological characteristics. However, in our previous studies, the frequency range was 3 kHz [22]. Because this frequency range is mostly used during the measurement of electrophysiological parameters, it is employed in electrophysiological tests and is less likely to interfere with the signal being monitored. The membrane potential, a crucial variable in electrophysiological investigations of plants, may also be precisely measured within this frequency range. Fresh branches’ youngest leaves, located in the fourth and fifth leaf positions, were chosen as the experimental material. Each leaf was clipped to the homemade, custom-made parallel-plate capacitors with a diameter of 10 mm Figure 1. The relationship curve between C, R, and Z with CF was established using Sigma plot (ver. 12.5, Systat Software, Inc., San Jose, Cal., CA, USA). The relationship between C, R, and Z with CF was fitted, and the model parameters were estimated.

Finally, the C, R, and Z at F = 0 could be calculated. Moreover, the same weight and quality of iron block were used, and finally, leaf XC and XL were calculated, respectively, according to Formulas (1) and (2):

(1) $$XC\;=\frac{1}{2\pi fC}$$

(2) $$\frac{1}{-XL}=\frac{1}{Z}-\frac{1}{R}-\frac{1}{XC}$$

where XC = capacitive reactance, π = 3.1416, f = frequency, C= physiological capacitance, XL = inductive reactance, Z = impedance, R = resistance.

### 2.4. Determination of Inherent Electrophysiological Parameters in Mangrove Leaves

The concentrations of electrolytes are associated with the outer and inner sides of the cell membrane, which are defined by plant leaf Z. The outside stimulation changes the membrane permeability of electrolytes, which impacts the outer and inner sides of the cell membrane. Bioenergetics says that the Nernst equation can be used to find the potential of ion groups and electric dipoles inside and outside the cell membrane [27]. Thus, the Nernst equation can be obtained from Equation (3) as follows:

(3) $$E-E^{0}=\;\frac{R_{o}T}{n_{z}F_{o}}ln\frac{Qi}{Qo}$$

where E: the electromotive force (V), $E^{0}$: the standard electromotive force (V), $R_{o}$: the gas constant (8.314570 J K^−1^ mol^−1^), T: the thermodynamic temperature (K), Qi: the concentration of electrolytes responding to Z inside the cell membrane (mol L^−1^), Qo: the concentration of electrolytes responding to Z outside the cell membrane (mol L^−1^), F_0_: Faraday constant (96,485 C mol^−1^), and $n_{z}$: the number of transferred electrolytes (mol).

Internal energy of electromotive force changed into pressure work, the relationship is directly proportional, PV = a E:

(4) $$PV=\;aE\;=\;aE^{o}+\frac{aR_{o}T}{n_{z}F_{o}}ln\frac{Qi}{Qo}$$

where P: the pressure intensity on the leaf cells (Pa), a: the energy conversion coefficient of the electromotive force, and V = the cell volume (m^3^). Further, $P=\frac{F}{S}$ where F: the clamping force (N) and S: the effective area of the electrode plate (m^2^). F can be calculated by the gravity formula:

(5) $$F=\;\left(M\;+\;m\right)g$$

where M: the iron block mass (kg), m: the mass of the plastic rod and the plate electrode (kg), and g: 9.8 N/kg.

For mesophyll cells, the sum of Qo and Qi is certain. Qi is directly proportional to the conductivity of electrolytes that respond to Z, and the conductivity is the reciprocal of Z. Hence, $\frac{Q_{i}}{Q_{o}}$ can be expressed as $\frac{Q_{i}}{Q_{o}}=\;\frac{\frac{J_{o}}{z}}{Q-\frac{J_{o}}{z}}=\;\frac{J_{o}}{QZ-J_{o}\;}$ where J_O_: the ratio coefficient of the conversion between Qi and Z, and Q_T_ is Qo + Qi. Therefore, Formula (2) can be transformed into Equation (6):

(6) $$\frac{V}{S}F=aE^{o}-\frac{a\;R_{o}T}{n_{z}F_{o}}ln\frac{Q_{T}Z\;-\;J_{o}}{J_{o}}$$

(7) $$\frac{a\;R_{o}T}{n_{z}F_{o}}ln\frac{QZ-J_{o}}{J_{o}}=aE^{o}-\frac{V}{S}F$$

And

(8) $$ln\frac{Q_{T}Z-J_{o}}{J_{o}}=\frac{n_{z}F_{o}E^{o}}{RT}-\frac{{Vn}_{Z}F_{o}}{S\;a\;RT}F$$

The exponents of both sides in Equation (9):

(9) $$\frac{Q_{T}Z\;-\;J_{o}}{J_{o}}=e^{\frac{n_{z}F_{o}{E^{o}}_{o}}{R_{o}T}}e^{\left(-\frac{{Vn}_{Z}F_{o}}{S\;a\;R_{o}\;T}F\right)}$$

Further,

(10) $$Z=\frac{J_{o}}{Q_{T}}+\frac{J_{o}}{Q_{T}}{\;e}^{\frac{n_{z}F_{o}E^{o}}{Ro\;T}}e^{\left(-\frac{{Vn}_{Z}Fo}{Sa\;Ro\;T}F\right)}$$

As $d=\frac{v}{s}$, Formula (10) is transformed into

(11) $$Z=\frac{J_{o}}{Q_{T}}+\frac{J_{o}}{Q_{T}}{\;e}^{\frac{n_{Z}FoE^{o}}{RoT}}e^{\left(-\frac{{dn}_{Z}Fo}{a\;Ro\;T}F\right)}$$

For the same leaf tested in the same environment, the d, a, E°, $R_{o}$, T, $n_{z}$, $F_{o}$, Q, and $J_{o}$ of Formula (10) are constants. Let $y_{o}=\frac{J_{o}}{Q},\;k_{1}=\frac{J_{o}}{Q}$ $e^{\frac{nzFoE^{o}}{RoT}},$ and $b_{1}=\frac{dn_{z}F_{o}}{aRoT}$; then, the intrinsic mechanical relationships of leaf Z and F are

(12) $$Z=y_{o}+k_{1}e^{-b_{1}F}$$

where $y_{o}$, k_1_, and b_1_ are the model parameters.

When F = 0, the inherent impedance (IZ) can be determined by

(13) $$IZ=y_{o}+k_{1}$$

Same as the Z, the inherent mechanical relationships of leaf X_C_ and F are revealed:

(14) $$Xc=p_{o}+k_{2}\;e^{-b_{2}F}$$

where $p_{o}$, $k_{2}$, and $b_{2}$ are the model parameters. When F = 0, the intrinsic capacitive reactance (IX_C_) (IRe) of the plant leaves can be calculated as follows:

(15) $$IXc=p_{o}+k_{2}$$

Like R, the inherent mechanism relation of leaf XL and F are calculated as follows:

(16) $$XL=q_{o}+k_{3}\;e^{-b_{3}F}$$

where $q_{o}$, k_3_, and b_3_ are model parameters. When F= 0, the inherent inductive reactance (IXL) of plant leaves could be calculated as:

(17) $$IXL=q_{o}+k_{3}$$

Thus, the inherent impedance (IZ) and inherent capacitance (IC) of plant leaves were obtained according to Equations (18) and (19):

(18) $$\frac{1}{IZ}=\frac{1}{IR}-\frac{1}{IXcR}-\frac{1}{IXL}$$

The clamping forces according to the first law of thermodynamics were obtained as follows:

(19) $$IC\;=\frac{1}{2\pi fIXc}$$

### 2.5. Nutrient Plunder Capacity of A. corniculatum and K. obavata Species

The inherent electrical parameters IR, IZ, IX_C_, IXL, IC, and ET were obtained by a mechanical equation, and based on these electrical parameters, the salt transport parameters were achieved. Details are mentioned in [24]. The nutrients flux per unit area (UNF), the nutrients transfer rate (NTR), and nutrients transport capacity (NTC) could be represented by Equations (20)–(22).

As we know, the cell membrane proteins are most closely related to the nutrients transport; thus, the relative nutrients flux per unit area (UNF) could be represented by Equation (20):

(20) $$UNF=\frac{IR}{IXc}+\frac{IR}{IXL}$$

Given that salts may dissolve in water and that the concepts of the water transfer rate and the nutrients transfer rate (NTR) are conceptually equivalent and given the same value, Equation (21) can be used to compute it:

(21) $$NTR=\frac{\sqrt{(I{C)}^{3}}}{IC\;xIZ}$$

Therefore, the nutrients transport capacity (NTC) is UNF multiplied by NTR:

*NTC* = *UNF* × *NTR* (22)

The surface and binding proteins on the cell membrane strongly influence the transport and metabolism of nutrients. The binding protein is most closely related to the active transport (or plunder capacity) of nutrients. Thus, the active transport (or plunder capacity) flow of nutrients (NAF) could be represented by Equation (23):

(23) $$NAF=\frac{IXC}{IXL}$$

Therefore, the nutrients plunder capacity (NPC) could be monitored by NAF multiplied by NTR.

NPC = NAF × NTR(24)

### 2.6. Photosynthetic Traits

The photosynthetic parameters such as PN, LSC, LCi, and LTr were measured utilizing a portable LI6400XT photosynthesis measurement system (LI-COR, Lincoln, NE, USA). The leaves of both species, *A. corniculatum* and *K. obavata,* were enclosed within the chamber for analysis. Each parameter was experienced with five replicates. The fully expanded youngest leaf from the top of the plant was selected for all measurements, and the experiment took place during full sunshine from 9:00 a.m. to 11:30 a.m. [28]. Throughout the data recording process, the following set values were applied: atmospheric pressure at 99.9 kPa, a flow rate inside the chamber of 500 µmol s^−1^, and photosynthetic active radiation (PAR) at 800 µmol m^−2^ s^−1^, using its own red and blue light source, and a reference CO_2_ concentration of 400 µmol mol^−1^

### 2.7. Growth Rate

Leaf area (cm) was determined by an allometric model equation with the help of the measuring scale and leaf area estimation, following Analuddin et al. (2009) [29]:

U = 0.8436 × (L × W)^0.9502^ (25)

where L was exposed to the maximum leaf width, W was exposed to the maximum leaf length in cm, and U was denoted the leaf area in cm^2^. The growth rate was calculated using a three-parameter logistic equation, the details of the equation are mentioned.

The logistic growth equation with three parameters (Equation (26)) was used for fitting plant height or leaf length versus number of days from the start of experiment for different species.

(26) $$u=\frac{U}{{1+k.e}^{-\lambda .t}}$$

where U, λ, and k are the maximum leaf area, intrinsic rate of increase, and constant, respectively. Assuming Equation (26), the half expansion period t*, which is the time necessary for the leaf to reach one half its U, defined as follows:

(27) $$t^{*}\;=\;\frac{lnk}{\lambda }$$

### 2.8. Statistical Analysis

The examination of the significant changes among all different salt stress treatments was subjected to analysis of variance (ANOVA) in SPSS software (version 20.0.) using Duncan’s multiple variable tests at *p* < 0.05. A correlation matrix of the study was based on Pearson’s correlation coefficients, using * and ** to show the *p* < 0.05 and *p* < 0.01 probability levels, respectively. The figures were prepared by Origin Pro. 9.0 (Northampton, MA, USA).

## 3. Results

The fitting equation between leaf Re, XC, XL, and CF of both mangrove species is shown in Table 1. These equations are chosen randomly from the list, and the results of fitting show a positive correlation between Re, XC, and XL with CF. All values of R^2^ are ≥0.98, and *p* values are <0.0001. The positive correlation highlighted the inherent relationship between Re, XC, and XL with the CF mechanism and their authentic existence.

### 3.1. Inherent Electrophysiological Parameters of Both Mangrove Species

The inherent electrophysiological characteristics of *A. corniculatum* species and *K. obavata* species are presented in Table 2. In *A. corniculatum*, leaf IRe, IX_C_, IXL, IZ, and IC displayed significant responses to salinity. Specifically, IRe, IXL, and IC values increased, whereas IX_C_ and IZ decreased under low (100 mM) and medium (250 mM) NaCl levels. The IRe of *A. corniculatum* also exhibited significant changes across the full range from low to high salinity (100–400 mM). In contrast, the IRe of *K. obavata* specie remained statistically unchanged at medium and high salt levels. Also, *A. corniculatum* species, IX_C_ and IXL, showed significant differences at low salt levels compared to the control, while *K. obavata* species vacuole volume is bigger compared to *A. corniculatum* species. Therefore, the IC was higher for *K. obavata* species in all salt treatments. The lowest IC was observed in *K. obavata* under high salt levels compared with (Table 2).

### 3.2. Nutrients Transport Parameters

**Figure 2:**
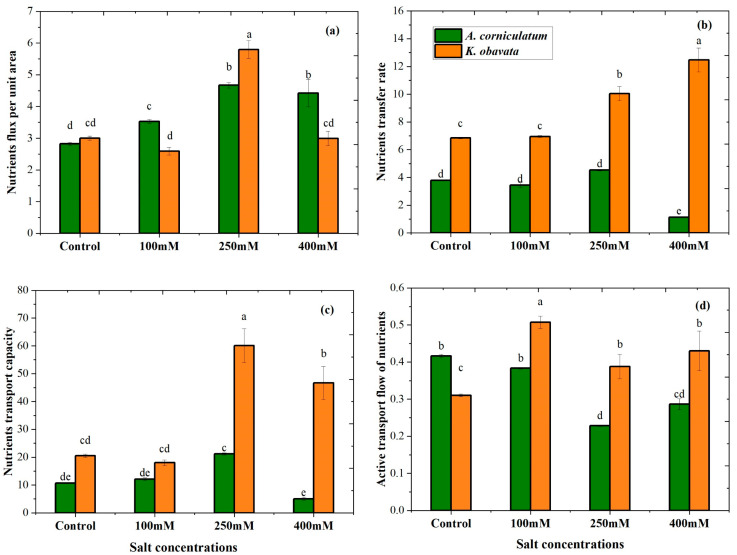
The effect of salt stress on *A. corniculatum* and *K. obavata* nutrients transport parameters. Note (**a**) UNF = nutrient flux per unit area; (**b**) NTR = nutrient transfer rate; (**c**) NTC = nutrient transport capacity; and (**d**) (NAF) = active transport flow of nutrients. Values represent means ± SE of five replicates (n = 5). Different lowercase letters indicate significant differences among treatments (*p* < 0.05).

Significant differences (*p* < 0.05) were recorded for the nutrient transport parameters of both species, as shown in Figure 2. Nutrient transport parameters were obtained based on a mechanistic equation. The results of UNF varied with changes in salt level; at the medium salt level, *A. corniculatum* and *K. obavata* species showed higher values compared to the control. Furthermore, NTR indicates that distinct responses between the two species; *K. obavata* consistently showed higher NTR values across low-, medium-, and high-salt treatments, in contrast to *A. corniculatum* which exhibited lower NTR values (Figure 2b). Notable results were observed for NTC at control and low salt levels; non-significant NTC values were found for both species, suggesting that both species can easily resist low salinity stress. However, the *K. obavata* species showed a greater change in NTC than the *A. corniculatum* species at medium salt level which increased by 241% and high salt level by 127% compared to the control shown in Figure 2c. The results of *A. corniculatum* showed a slight reduction (–7.9%) under low salinity, followed by sharp decreases under medium (–45.1%) and high (–31.2%) treatments. In contrast, *K. obovata* displayed strong positive responses, with nutrient flow increasing by 63.7% at low salinity, 25.1% at medium salinity, and 38.7% at high salinity relative to the control (Figure 2d).

### 3.3. Nutrients Plunder Capacity

**Figure 3:**
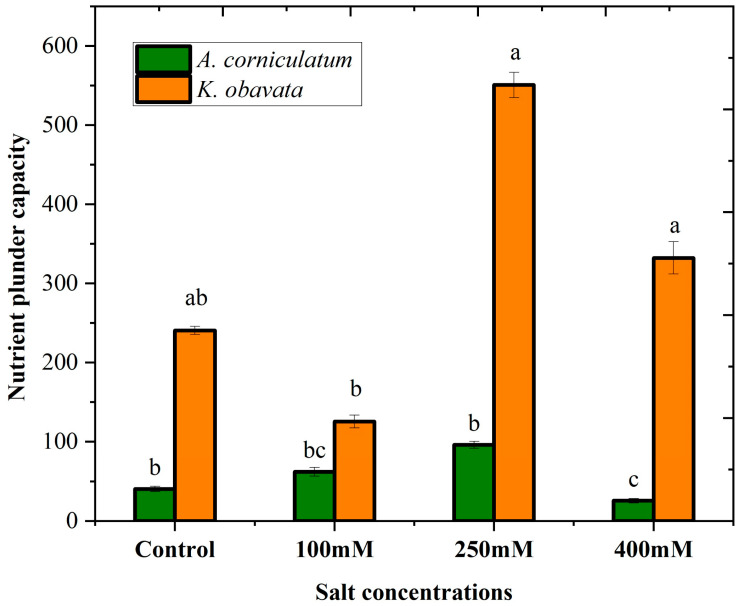
Influence of different salt stress treatments on the nutrient plunder capacity of two mangrove *A. corniculatum* and *K. obavata* species. Values represent means ± SE of five replicates (n = 5). Different lowercase letters indicate significant differences among treatments (*p* < 0.05).

Various salt concentration treatments affected the nutrient plunder capacity (NPC) of both species, as shown in Figure 3. Both species showed a marked difference in NPC values, while *A. corniculatum* species values increased by 3.65% and 136% at low and medium salt levels compared to the control, but decreased by 85.4% at high salt levels. In contrast, *K. obavata* specie obtained substantially higher NPC values at medium and high salt levels by 505% and 421% increases, respectively, compared to the control. However, at low salt level values, they decreased by 10.7% compared to the control treatment.

### 3.4. Photosynthetic Parameters

**Figure 4:**
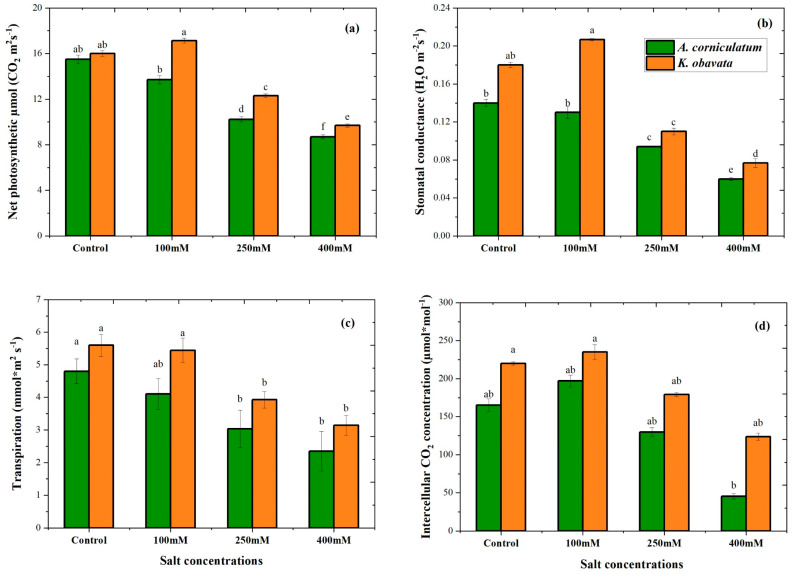
Impact of different salt stress treatments on photosynthetic parameters of *A. corniculatum* and *K. obavata* species. (**a**) net photosynthesis (PN), (**b**) stomatal conductance (LSC), (**c**) transpiration (LTR), and (**d**) intracellular CO_2_ (LCi). Values represent means ± SE of five replicates (n = 5). Different lowercase letters indicate significant differences among treatments (*p* < 0.05).

Figure 4 illustrates the effects of different salt stress treatments on photosynthetic parameters, including net photosynthesis, stomatal conductance, transpiration, and intracellular concentration. Both species showed significant differences under salt stress conditions. *A. corniculatum* indicates higher values for all parameters under low salt treatment as compared to the control. The PN values of both species decreased progressively with increasing salt concentration. The *A. corniculatum* and *K. obovata* species exhibited significant decreases in PN values of 43% and 39%, respectively, at high salt levels compared to the control. Stomatal conductance results indicate that both species performed well at low salt levels. Specifically, *K. obovata* increased stomatal conductance by 14% under the low salt levels, while *A. corniculatum* showed a 7% reduction in stomatal conductance under the same conditions. Both species exhibited a similar reduction in stomatal conductance by 57% under high salt levels compared to the control. Regarding leaf transpiration, *A. corniculatum* showed a 14% reduction in the low salt level, while *K. obovata* showed a 2% reduction compared to the control. However, a more pronounced reduction in transpiration was observed in both species under high salt stress.

### 3.5. Allometric Curve Fitting Growth Model

**Figure 5:**
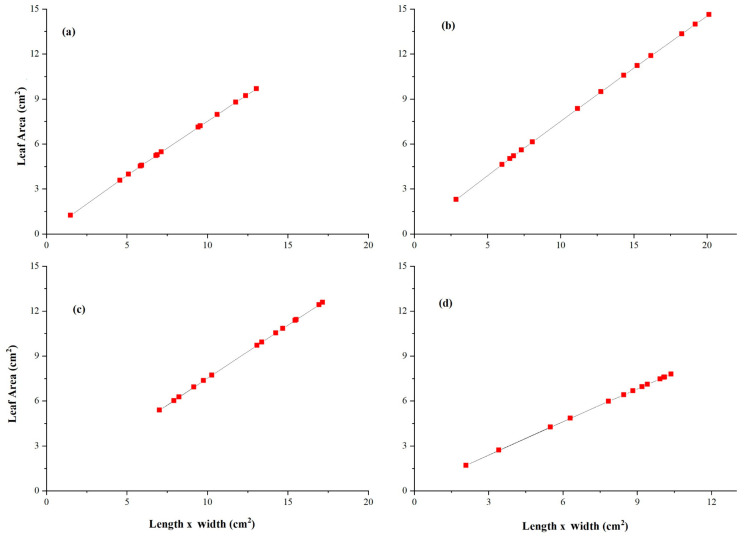
Allometric relationship of leaf area u to length/times width w with influence of different salt (NaCl) treatment (**a**) Control, (**b**) 100 mM, (**c**) 250 mM, and (**d**) 450 mM of *A. corniculatum* species. The straight line given by Equation (26).

**Figure 6:**
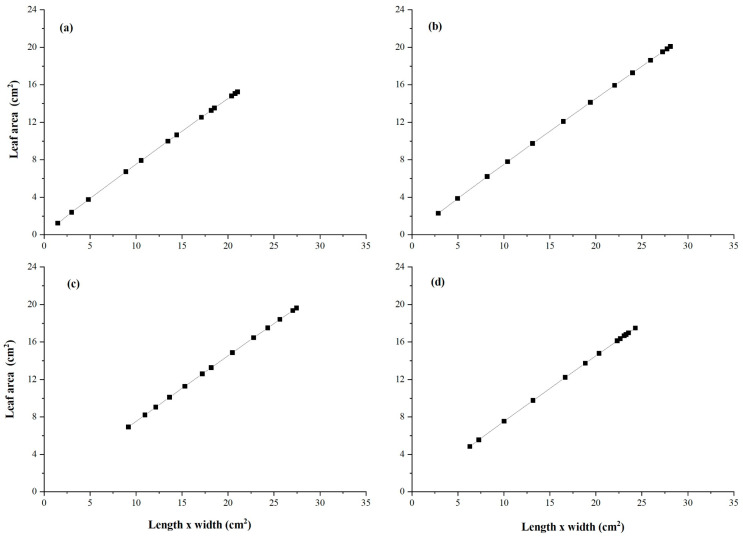
Allometric relationship of leaf area u to length/times width w with influence of different salt (NaCl) treatment (**a**) Control, (**b**) 100 mM, (**c**) 250 mM, and (**d**) 450 mM of *K. obavata* species. The straight line is given by Equation (25).

The fitting curve follows Equation (26) for both species. Figure 5 shows the fitting curve of *A. corniculatum* under different salt levels, including the control treatment. The value of R^2^ = 0.99 for all treatments indicates that the allometric growth model fits the *A. corniculatum* species well. Moreover, Figure 6 shows the fitting curve of *K. obavata* species under various salt levels with control treatments. The value of R^2^ = 0.99 for all treatments indicates that the allometric growth model fits *K. obavata* species well.

### 3.6. Leaf Growth Rate

**Figure 7:**
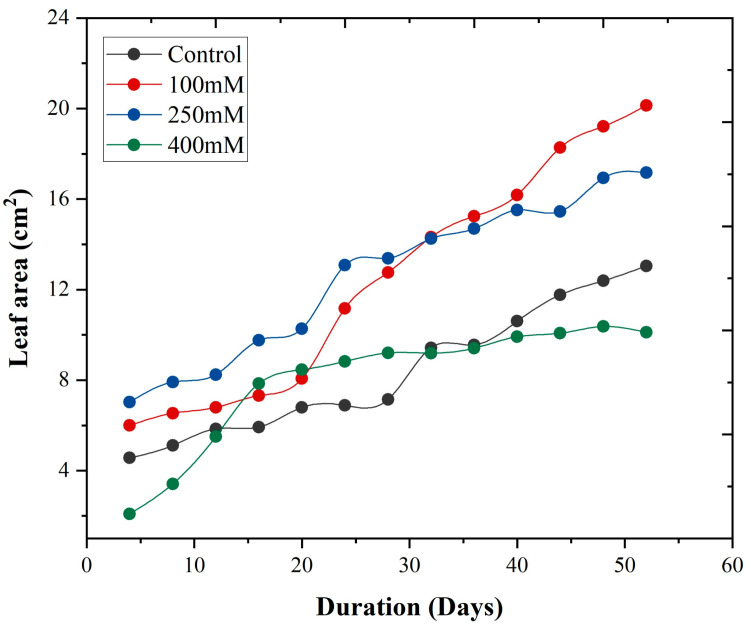
Leaf area growth with respect to time t (days) in different salt treatments with influence of different salt (NaCl) treatment of *A. corniculatum* species. The curves are given by Equation (26).

Figure 7 illustrates the growth rate of *A. corniculatum* species under different NaCl treatments over time (days). It was observed that the growth rates at low and medium salt levels were significantly higher compared to the control treatment. In contrast, a notably lower growth rate was observed at high salt levels. This indicates a clear trend where increasing salt concentration results in a decreased growth rate for *A. corniculatum*. The data clearly show that *A. corniculatum* species are more tolerant to low and medium salt levels, but their growth is adversely affected by higher salt concentrations.

**Figure 8:**
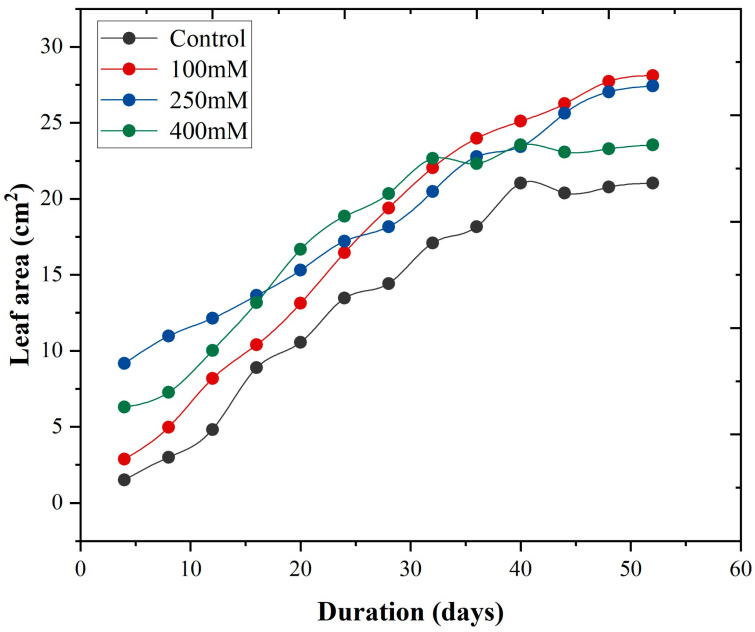
Leaf area growth with respect to time t (days) in different salt treatments with influence of different salt (NaCl) treatments of *K. obavata* species. The curves are given by Equation (26).

Figure 8 illustrates the growth rate of *K. obavata* species under different NaCl treatments over time (days). The results show that the growth rates under low and medium salt levels are almost the same and higher than under control conditions. Additionally, the growth rate at high salt concentration is also higher than under control conditions, with a correlation value of R = 0.95. These findings demonstrate a clear difference in growth rates between the treatments, suggesting that medium and low salt levels are more suitable compared to the control treatment.

### 3.7. Three Logistic Growth Parameter Equations

**Figure 9:**
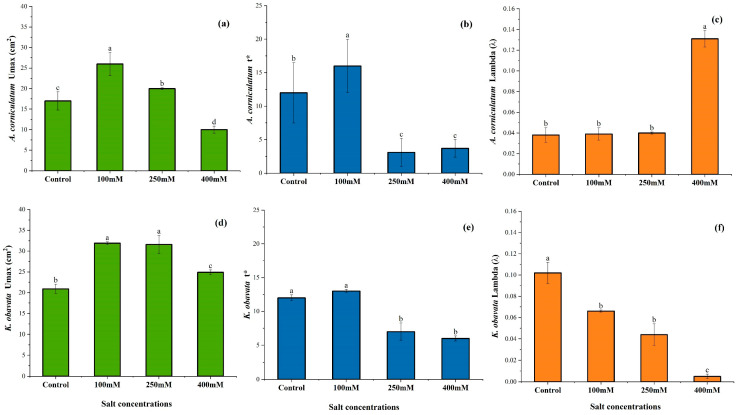
Different time variation in maximum (**a**) leaf area Umax (cm^2^), (**b**) half expansion period t* and (**c**) intrinsic rate increase λ of *A. corniculatum*, while (**d**) leaf area Umax (cm^2^), (**e**) half expansion period t* and (**f**) intrinsic rate increase λ of *K. obavata* species. Values show as mean ± SE of five replicates (n = 5). The same letters stand for no significant difference at a significant level of 0.05.

Figure 9a–c shows the statistical analysis of maximum leaf area Umax (cm^2^), half expansion period (t*), and intrinsic rate of increase (λ) for *A. corniculatum* species. The results for Umax showed a significant difference across the treatments, with higher values at low salt levels and lower values at high salt levels compared to the control. The t* results are notable; the high salt level showed greater values, while the other treatments have almost the same values. This indicates that when the species is exposed to high salt levels, its growth increases sharply initially but decreases over time. The λ results were significant, with higher values obtained at low salt levels compared to other treatments. Moreover, Figure 9d–f present the statistical analysis of Umax, t*, and λ for *K. obavata* species. The results Umax indicate a significant difference among treatments, with non-significant differences observed at low and medium salt levels. Furthermore, the results t* are intriguing; in the control treatment, higher values were observed, while lower values were observed at high salt levels. These values changed over time, as reflected in the growth rate parameters. The λ results were significant, with higher values obtained at low salt levels and lower values seen at high salt levels compared to other treatments.

## 4. Discussion

The current study examines the electrophysiological parameters of two mangrove species. These parameters play an essential role in plant life cycle activities. The electrophysiological parameters give more accurate responses as compared to photosynthesis parameters when plants face stress conditions [27]. However, in this study, photosynthesis parameters are used only as supportive parameters. Further, the current study focuses on two important parameters, including active transport flow of nutrients (NAF) and nutrient plunder capacity (NPC), based on plant electrophysiological characteristics. Electrical parameters responded more rapidly in high-stress conditions compared to photosynthesis parameters. The presented results of fitting equations of Re-CF, XC-CF, and XL-CF had good correlations; all values of R^2^ were ≥ 0.98, and *p* values were < 0.0001. The positive correlation highlighted the inherent relationship between Re-CF, XC-CF, and XL-CF mechanisms and their authentic existence, as shown in Table 1. The inherent parameters are connected to plant cells and their vacuoles. The concentration of electrolytes on the inner and outer sides changes in the cell membrane, which leads to variation in leaf C, Re, and Z. The results of leaf IRe, IX_C_, IXL, IZ, and IC of *A. corniculatum* species demonstrated significant value increases in IRe, IXL, and IC, as well as IX_C_ and IZ value decreases, with increasing salt concentrations, as shown in Table 2. The results of the present study showed that *K. obavata* species had greater salt tolerance ability than *A. corniculatum*. A previous study supported the current study results that *K. obavata* species’ vacuole size is larger; therefore, it stores more salt even at high salt levels [4]. Although both species showed significant values of electrical parameters, they deal with salt stress in different ways and respond to stress conditions. For example, *K. obavata* species have a larger cell volume than *A. corniculatum* species as previous research states that IC values directly link with vacuole capacity in plant cells [24]. *A. corniculatum* species have small vacuoles that utilize different mechanisms, such as salt excretion and salt exclusion [30]. Another study also reported that *A. corniculatum* and *K. obavata* have different mechanisms for salt stress tolerance [17,31]. The cell volume is directly correlated with vacuoles, water is a major factor for vacuole and cytoplasm, and the mechanism of vacuolar size is directly associated with plant CP. In our recent study, it was noted that salt outflow capacity and salt ultrafiltration capacity improved at low salt levels in *A. corniculatum* species, and high salt level re-watering techniques improved the salt outflow capacity and salt ultrafiltration of *K. obavata* species [24]. It was proved that when the salt level decreased, *A. corniculatum* species could not reverse, but *K. obavata* species could reverse their actual situation. The results of nutrient transport parameters of both species are shown in Figure 3. The UNF results exhibited variations with salt levels, with higher values observed at the medium salt level compared to the control. while *A. corniculatum* species demonstrated higher values at high salt levels. However, other transport parameters of both species showed *K. obavata* performed well compared to the *A. corniculatum* species. A former study reported that nutrient metabolism and the overall development of plants are strongly associated with nutrient transport or plunder capacity, and plants have different adaptive mechanisms to low-nutrient environments [32].

Moreover, according to previous findings that support the current study, V = $\alpha \sqrt{{\mathrm{CP}}^{3}}$, volume of vacuole (V) and physiological capacitance (CP), so both are directly linked with each other if the physiological capacitance is higher, which states that the size of the vacuole is bigger [4]. The results of the NPC for both species are illustrated in Figure 4. *A. corniculatum* exhibited non-significant results at low and medium salt levels, while NPC values decreased at the high salt level compared to the control. In contrast, *K. obavata* species obtained higher NPC values at both medium and high salt levels, with lower values observed at the low salt level compared to the control. Overall, the NPC results were higher for *K. obavata* species than for *A. corniculatum.*

These results indicate that *K. obavata* species performed greater and could adapt to the saline soil environment of the coastal estuary wetland [29]. In previous results, there was a detailed discussion about three different capacities (salt outflow capacity, salt dilution capacity, and salt ultrafiltration capacity), which depend on the salt-resistant capacity of mangrove species, so it was noticed that for the low-saline area, *A. corniculatum* species is suitable, and for the high-saline area, *K. obavata* species is more suitable because it resists more salt [24]. *A. corniculatum* exhibited decreasing PN values as the salt concentration increased compared to the control (Figure 4a). In the comparison between both species, *K. obavata* obtained higher PN values than *A. corniculatum*. In a prior investigation, it was observed that elevated salinity led to a decline in photosynthesis due to the closure of LSC and a reduction in CO_2_ assimilation, identified as the primary factor causing salt-induced stress on photosynthetic activity [33]. The results of LSC for *K. obavata* species were particularly interesting, as LSC values exhibited a continuous decrease with increasing salt concentration (Figure 4b). The change in water content due to the opening and closing of LSC triggers an imbalance of gaseous exchange [34]. Additionally, the values of LTr for *K. obavata* species were higher compared to *A. corniculatum*. In both species, LTr values decreased, and non-significant results were observed in the control and low salt levels (Figure 4c). The rates of both CO_2_ assimilation and LSC exhibited a decrease with an increase in environmental salinity, as evidenced in another study [35]. Consistent findings were reported in a mangrove study, indicating that heightened salt stress impeded photosynthesis by inducing stomatal closure [36].

The results of LCi showed that both species obtained higher values at low salt levels. However, non-significant results were noted at medium and high salt levels. Across all photosynthetic parameters, *K. obavata* species consistently obtained higher values than *A. corniculatum (*Figure 4d). These findings align with a previous study that indicated that the *K. obavata* species can thrive when the salt concentration reaches 500 mM NaCl [31]. Another study also reported that *K. obavata* species tolerate chilling stress well compared to *A. corniculatum* species [37].

In Figure 5 and Figure 6, the results of the growth model for both mangrove species are presented. The fitting curve of both mangrove species shows that under different treatments and allometric model fits for both species, the value of R^2^ = 0.99. Moreover, the growth rate of leaf area, as shown in Figure 7, further elucidates the response of *A. corniculatum* to different salt treatments. These results clearly show that *A. corniculatum* is more tolerant to low and medium salt levels, but its growth is adversely affected by higher salt concentrations. When exposed to high salt concentrations, the growth rate decreases over time. A former study monitoring growth rate stated that the growth rate decreased in proportion to decreased density and mortality rate [38]. In comparison, the results for *K. obavata* species, presented in Figure 8, indicate that the growth rates at medium and low salt levels are higher than those under control conditions. Even at high salt levels, the growth rate is still higher than under control conditions. This is because *K. obavata* is a more salt-tolerant species and can withstand high-stress conditions effectively.

A research study reported that leaf growth increases more in the winter season as compared to summer [29]. In a former study, it was stated that photosynthesis and plant growth rate had a strong relationship [39,40]. Another study also reported that *K. obavata* species growth rate is the same in 30 days in low salt levels compared to medium salt levels [41]. Growth rate and nutrient parameters can be determined simultaneously, as growth rate is inherently linked to certain parameters. The growth rate also concerns LSC; lower LSC minimizes the leaf thickness and decreases the growth rate [17]. This similarity was seen in the previous study by Hwang et al. [42]. The half-expansion period t* and intrinsic rate of increase (λ) for *A. corniculatum* species are shown in Figure 9a–c. High salt levels obtained higher values compared to other treatments. These results indicate that the half-expansion period growth was sharply increased, but it decreased with respect to time. Furthermore, the intrinsic rate of increase shows high values at low salt levels as compared to the control treatment. Moreover, in Figure 9d–f, the half-expansion period for *K. obavata* species shows that the highest values were obtained in the control treatment. This indicates that the growth rate of *K. obavata* species initially increased sharply under control but decreased after some time. In summary, the differential responses of *A. corniculatum* and *K. obovata* to salt stress highlight species-specific strategies in nutrient transport and photosynthetic regulation. These findings provide new evidence that electrophysiological parameters can serve as reliable indicators of active ion transport in mangroves. Importantly, this approach offers a rapid and non-destructive method that may be extended to other halophytes for evaluating salt tolerance mechanisms under changing coastal environments.

## 5. Conclusions

This study provides new insights into the nutrient plunder (active ion transport) capacity of two mangrove species, *A. corniculatum* and *K. obovata*, by analyzing their electrophysiological response under different salt concentrations. This method presents a novel, rapid, and reliable approach for quantifying ion transport in mangroves. The active transport flow of nutrients and nutrient plunder capacity of mangrove species were evaluated for the first time using inherent electrical parameters. The NAF values were higher at low salt levels in *A. corniculatum*, reflecting its tolerance to less saline conditions. However, its NPC declined significantly under high salinity stress. In contrast, *K. obovata* demonstrated greater NPC at medium and high salt concentrations, highlighting its enhanced adaptability to saline soil environments. These results suggest that *K. obovata* may be better suited for restoration or conservation efforts in high-salinity coastal ecosystems. Future research should extend this approach to a broader range of mangrove species to further elucidate species-specific ion transport mechanisms under salt stress. In addition, integrating long-term field studies with controlled experiments, as well as combining physiological and molecular analyses, will provide deeper insights into adaptive strategies. Such investigations will not only improve restoration planning but also enhance predictive models for mangrove resilience under projected climate-driven salinity shifts.

## Figures and Tables

**Table 1:** The fitting equation parameters of *A. corniculatum* and *K. obavata* Re: resistance, XC: capacitive reactance, XL: inductive reactance.

*Aegiceras corniculatum*									
	Re-F			XC-F			XL-F		
Branch Leaf	Y_0_/k_1_/b_1_	R^2^	*p*<	Y_0_/k_2_/b_2_	R^2^	*p*<	Y_0_/k_3_/b_3_	R^2^	*p*<
1-1	0.5/1.62/0.49	0.999	0.0001	0.17/0.71/0.45	0.999	0.0001	0.66/2.06/0.49	0.999	0.0001
1-2	0.4/2.20/0.62	0.997	0.0001	0.17/0.91/0.52	0.998	0.0001	0.58/2.73/0.59	0.997	0.0001
1-3	0.8/1.23/0.44	0.999	0.0001	0.31/1.01/0.47	0.998	0.0001	1.06/1.91/0.46	0.999	0.0001
3-1	0.2/2.14/0.65	0.999	0.0001	0.11/0.89/0.51	0.999	0.0001	0.36/2.66/0.61	0.998	0.0001
3-2	0.5/3.61/0.66	0.999	0.0001	0.19/1.10/0.55	0.994	0.0001	0.64/4.19/0.64	0.992	0.0001
3-3	0.7/3.71/0.47	0.999	0.0001	0.26/1.15/0.40	0.998	0.0001	0.88/4.35/0.46	0.996	0.0001
5-1	1.1/2.96/0.27	0.998	0.0001	0.32/1.28/0.40	0.996	0.0001	1.31/3.68/0.30	0.998	0.0001
5-2	1.7/6.37/0.53	0.996	0.0001	0.44/1.56/0.48	0.993	0.0001	2.02/7.21/0.52	0.995	0.0001
5-3	1.6/8.65/0.67	0.998	0.0001	0.37/1.78/0.52	0.995	0.0001	1.85/9.59/0.65	0.989	0.0001
*Kandelia obovata*									
1-1	0.09/1.09/0.63	0.992	0.0001	0.09/0.51/0.53	0.995	0.0001	0.16/1.38/0.59	0.993	0.0001
1-2	0.04/0.38/0.62	0.998	0.0001	0.06/0.55/0.59	0.997	0.0001	0.09/0.79/0.60	0.998	0.0001
1-3	0.02/0.61/0.35	0.999	0.0001	0.04/0.63/0.39	0.997	0.0001	0.05/1.05/0.37	0.999	0.0001
3-1	0.05/0.46/0.75	0.994	0.0001	0.07/0.98/0.87	0.996	0.0001	0.17/1.34/0.67	0.992	0.0001
3-2	0.10/0.88/0.71	0.990	0.0001	0.10/0.70/0.64	0.994	0.0001	0.18/0.99/0.60	0.992	0.0001
3-3	0.10/0.56/0.65	0.990	0.0001	0.11/0.60/0.56	0.994	0.0001	0.11/1.22/0.82	0.995	0.0001
5-1	0.63/4.58/0.53	0.999	0.0001	0.21/1.29/0.51	0.996	0.0001	0.75/5.19/0.52	0.998	0.0001
5-2	0.62/3.11/1.12	0.998	0.0001	0.17/3.22/0.57	0.999	0.0001	0.76/29.6/1.03	0.998	0.0001
5-3	0.19/3.46/0.97	0.990	0.0001	0.10/0.89/0.64	0.993	0.0001	0.26/3.75/0.88	0.990	0.0001

**Table 2:** Influence of different salt (NaCl) treatments on inherent electrophysiological resistance, IX_C_ = inherent capacitive reactance, IXL = inherent inductive reactance, IZ = inherent impedance, IC = inherent capacitance.

*Aegiceras corniculatum*					
Salt Concentrations (mM)	Ire (MΩ)	IX_C_ (MΩ)	IXL (MΩ)	IZ (MΩ)	IC (pF)
Control	3.43 ± 0.06 de	1.72 ± 0.05 c	4.13 ± 0.03 d	1.46 ± 0.01 c	30.8 ± 0.10 d
100	4.11 ± 0.04 cd	1.61 ± 0.01 cd	4.21 ± 0.05 d	1.67 ± 0.09 b	32.8 ± 0.24 cd
250	5.43 ± 0.08 b	1.43 ± 0.005 cd	6.26 ± 0.02 b	1.34 ± 0.01 d	37.2 ± 0.14 bc
400	11.64 ± 0.79 a	3.41 ± 0.15 a	11.9 ± 0.09 a	3.47 ± 0.002 a	15.6 ± 0.67 f
*Kandelia obavata*					
Control	3.01 ± 0.05 e	1.31 ± 0.006 d	4.22 ± 0.06 d	0.93 ± 0.006 e	40.4 ± 0.17 b
100	2.61 ± 0.10 e	1.52 ± 0.04 cd	3.01 ± 0.01 e	0.85 ± 0.005 e	34.8 ± 1.02 bc
250	3.96 ± 0.03 cd	0.95 ± 0.07 e	2.47 ± 0.02 f	0.62 ± 0.005 f	56.1 ± 3.95 a
400	5.54 ± 0.03 c	2.21 ± 0.27 b	5.14 ± 0.03 c	0.32 ± 0.005 g	24.6 ± 2.93 e

Note: Values indicate the mean ± SD, (n = 5). The small letter indicates a significant difference (*p* < 0.05) using Duncan’s multiple range tests.

## Data Availability

All data in this research can be obtained from the corresponding authors upon reasonable request. The data are not publicly available due to privacy or ethical restrictions.

## Supplementary Materials

The following supporting information can be downloaded at: https://www.mdpi.com/article/10.3390/plants14203228/s1, Figure S1: Experimental design showing three saline treatments and a control: Control (0 mM), low (T1, 100 mM), medium (T2, 250 mM), and high (T3, 450 mM). Two mangrove species were included in the experiment: *Aegiceras corniculatum* (L.) Blanco and *Kandelia obovata* Sheue, H.Y. Liu & J.W.H. Yong; Table S1: Monthly averages of maximum and minimum temperature and total rainfall during the experimental period; Figure S2: Illustrates the daily variations is maximum and minimum temperature from March to May. The data highlights the progressive increase in both maximum and minimum temperatures as the season advanced, providing an overview of the climatic conditions during the experiment.
