# Supplementary material for: Electrophysiological Regulation of Nutrient Transport in Mangrove Species Under Salinity Stress: A Comparative Physiological Analysis of Aegiceras corniculatum (L.) Blanco and Kandelia obovata Sheue, H.Y. Liu & J.W.H. Yong
Source: Plants (Basel). 2025 Oct 20;14(20):3228. doi: 10.3390/plants14203228 (PMC12566782; doi:10.3390/plants14203228)
Supplement: Supplementary file 1 [file plants-14-03228-s001.zip › plants-3904259-supplementary.docx]

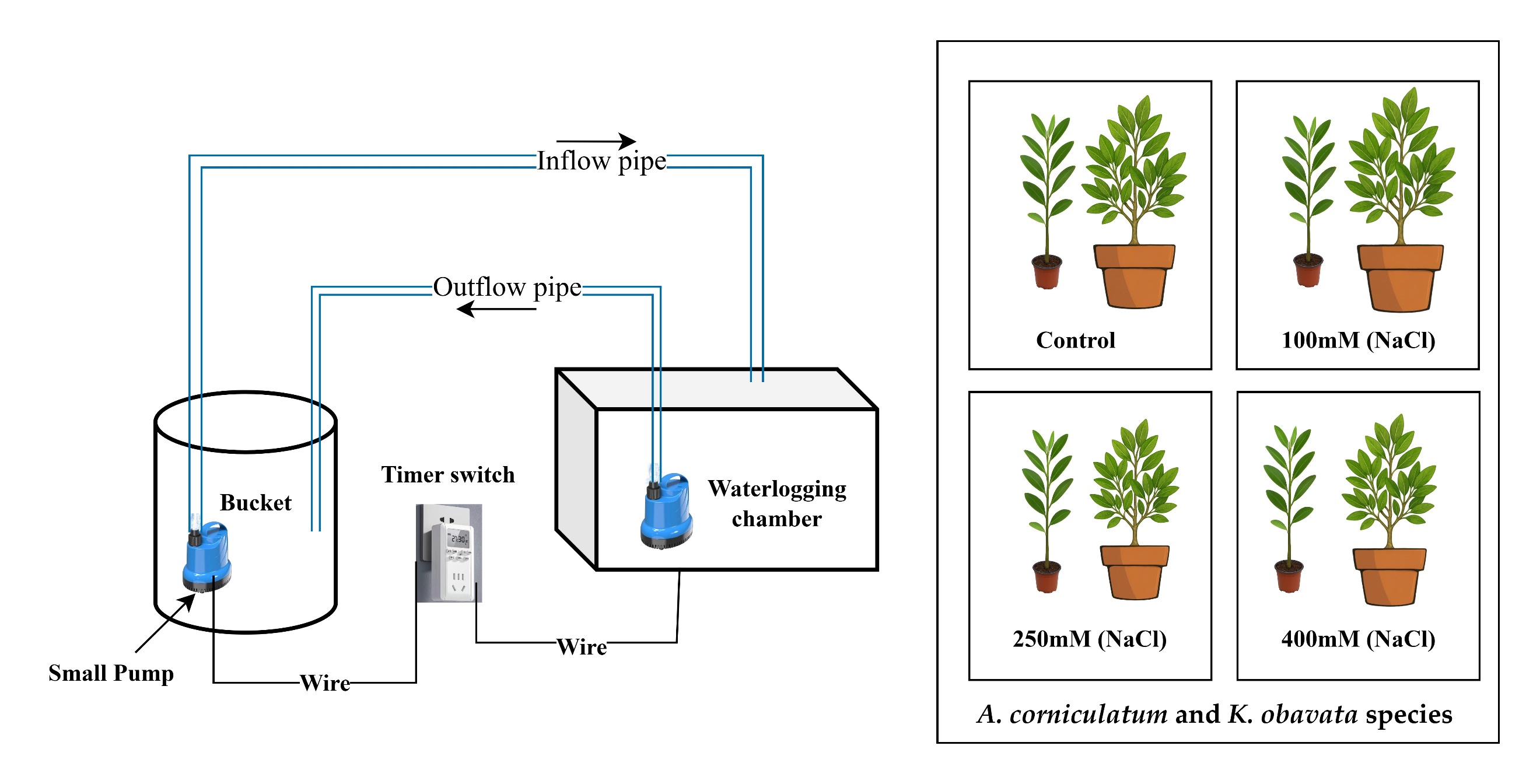


**Figure S1.** Experimental design showing three saline treatments and a control: Control (0 mM), low (T1, 100 mM), medium (T2, 250 mM), and high (T3, 450 mM). Two mangrove species were included in the experiment: *Aegiceras corniculatum* (L.) Blanco and *Kandelia obovata* Sheue, H.Y. Liu & J.W.H. Yong.

**Table S1:** summarizes the monthly climatic conditions recorded during the experimental period from March to May. The values include the average maximum and minimum temperatures along with the total rainfall for each month. The climatic data were obtained from Tianqi24 historical weather records for Zhenjiang ([https://www.tianqi24.com/zhenjiang/history2023.html](https://www.tianqi24.com/zhenjiang/history2023.html?utm_source=chatgpt.com))

**Table S1**. Monthly averages of maximum and minimum temperature and total rainfall during the experimental period.

| **Month** | **Maximum °C** | **Minimum °C** | **Total Rainfall (mm)** |
| --- | --- | --- | --- |
| March | 18 | 7 | 54.2 |
| April | 22 | 11 | 108.3 |
| May | 27 | 16 | 124.9 |

Figure S2 illustrates the daily variations in maximum and minimum temperatures from March to May. The data highlights the progressive increase in both maximum and minimum temperatures as the season advanced, providing an overview of the climatic conditions during the experiment. <https://tianqi.2345.com/wea_history/58248.htm>


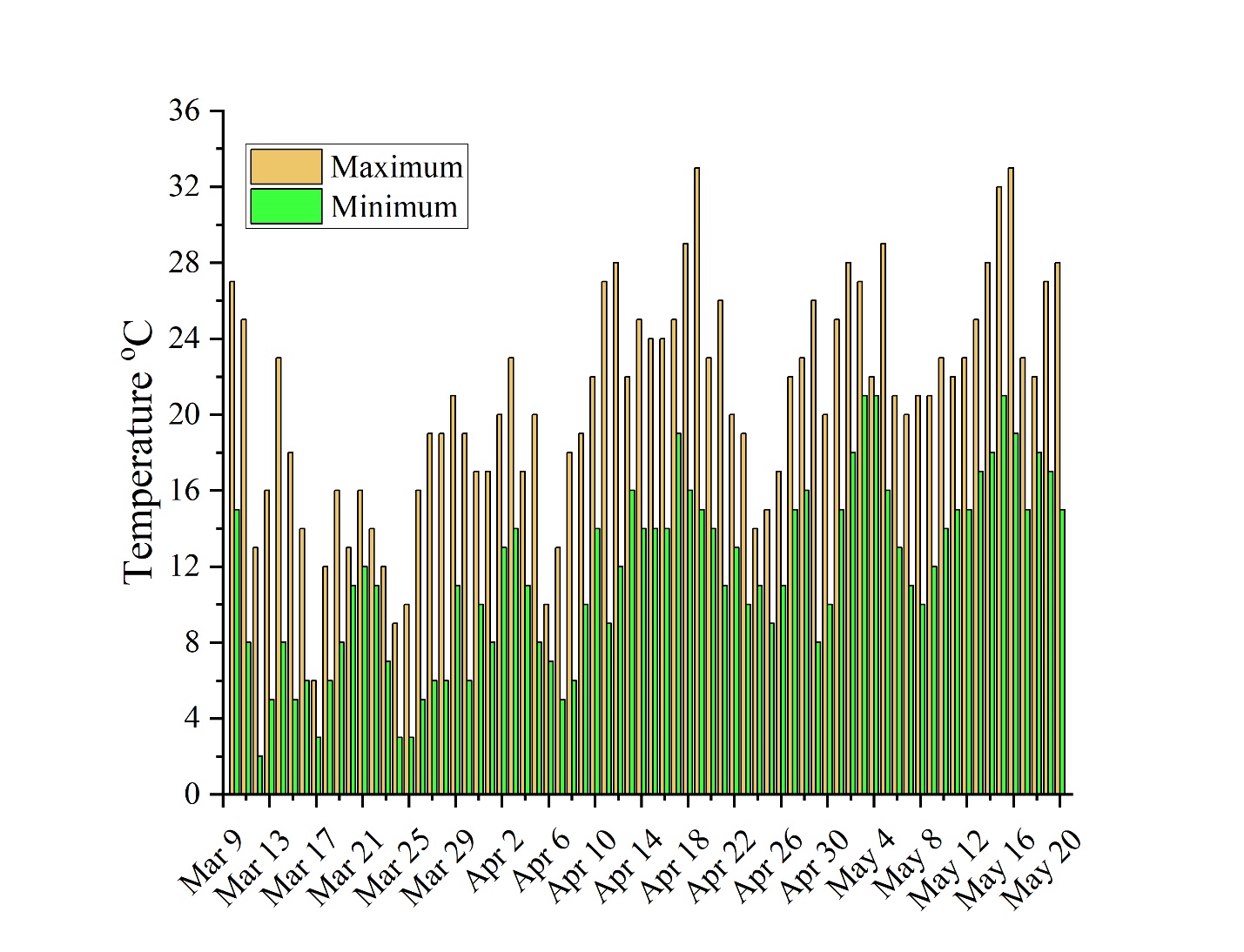
**Figure S2**. Daily maximum and minimum temperatures recorded during the experimental period.
